# Anti-NMDAR encephalitis in a child with long impaired consciousness and persistent antibodies: a case report and mini review

**DOI:** 10.3389/fimmu.2024.1402523

**Published:** 2024-05-28

**Authors:** Wenhao Zhang, Wenjia Cao, Wenhan Tao, Yufei Wang, Chenchen Tangzhu, Qinru Shen, Xulai Shi

**Affiliations:** Department of Pediatric Neurology, The Second Affiliated Hospital & Yuying Children’s Hospital of Wenzhou Medical University, Wenzhou, Zhejiang, China

**Keywords:** anti-NMDAR encephalitis, anti-NMDAR antibody, autoimmune encephalitis, case report, prognosis

## Abstract

We described a challenging case of anti-N-methyl-D-aspartate receptor (NMDAR) encephalitis in a young girl. Despite enduring months of reduced consciousness with ongoing antibody presence, she ultimately exhibited remarkable improvement within a 5-year follow-up period. Additionally, we conducted a concise review of relevant literature on anti-NMDAR encephalitis, with a specific focus on anti-NMDAR antibodies. Our findings enhance the clinical comprehension of anti-NMDAR encephalitis and offer valuable insights to clinicians for its management.

## Introduction

Since its initial description in 2007, anti-N-methyl-D-aspartate receptor (NMDAR) encephalitis has gained increasing recognition. It primarily affects women and has a median age of onset of 21 years (1). The typical course of anti-NMDAR encephalitis includes five stages: a prodromal phase, a psychotic and/or seizure phase, an unresponsive and/or catatonic phase, a hyperkinetic phase, and a gradual recovery phase (2). However, in children, particularly those under 13 years old, their presentations and progression often differ from those seen in young adults. Notably, the pediatric group exhibits a more balanced sex ratio and a weaker association with cancer (3). Children commonly present with seizures, abnormal movements, insomnia, and irritability. They are also more prone to progressing to multifocal neuropsychiatric symptoms rather than maintaining a single clinical syndrome (4). While still considered rare, anti-NMDAR encephalitis has emerged as a significant cause of pediatric encephalitis in certain regions, potentially attributed to increased awareness and accessibility of testing technology (5). This report describes a patient suffering from long impaired consciousness with persistent antibodies in her serum, but still achieve an impressive outcome.

## Case presentation

An 8-year-old previously healthy girl was admitted to our hospital due to altered mental status persisting for the previous 4 days, along with recurrent and relentless seizures. Upon arrival at the emergency department, despite receiving phenobarbitone and diazepam, her seizures persisted for nearly 2 h. Lumbar puncture revealed normal cerebrospinal fluid (CSF) pressure, along with 45 nucleated cells (90% lymphocytes), a protein level of 24 mg/dL, and a glucose level of 2.3 mmol/L. Initially, viral encephalitis was suspected based on the CSF findings. However, her seizures worsened after treatment initiation, and she became completely unresponsive with increased involuntary movements. Autoimmune encephalitis (AE) was then considered, and on the fifth day of hospitalization, serum testing using an NMDA-positive cell-based assay confirmed the presence of anti-NMDAR antibodies with a titer of 1:100. Unfortunately, CSF testing for anti-NMDAR antibodies was not performed. Subsequent routine electroencephalography (EEG) revealed abnormal slow wave and delta brush patterns, leading to a diagnosis of anti-NMDAR encephalitis. Brain magnetic resonance imaging (MRI) throughout her illness showed no abnormalities, and no evidence of tumor was detected. Herpes simplex virus (HSV) IgM antibodies were negative. Treatment with high-dose intravenous methylprednisolone (IVMP, 15 mg/kg/day for 3–5 days) and intravenous immunoglobulin (IVIG, 2 g/kg over 3–5 days) was initiated. Despite two courses of IVMP and IVIG within the first month, the patient showed no improvement, and serum antibody titer increased to 1:1,000. Meanwhile anti-NMDAR antibody titer in CSF was 1:100, but routine CSF analysis turned normal. Rituximab (375 mg/m^2^ weekly in 4 weeks) was then administered weekly in addition to prolonged IVIG and IVMP. Within a month, CD19 B-cell levels decreased to less than 0.1%, CSF antibody titer decreased to 1:32, and the frequency of seizures and involuntary movements noticeably decreased, but serum antibody titer increased to 1:1,000 and mental status improvement was negligible. Throughout her hospitalization, she experienced recurrent low fever with increased sputum production. Initially, her respiratory function appeared unaffected, but she later developed paroxysmal hypoxemia, with both frequency and severity gradually increasing. On the 50th day of hospitalization, her respiratory condition worsened, necessitating transfer to the pediatric intensive care unit (PICU) for assisted ventilation. Chest computed tomography scan revealed collapse of the right upper lobe and scattered infiltration. Sputum culture revealed the presence of *Pseudomonas aeruginosa*, for which imipenem/cilastatin was administered, along with oxygen delivered at a rate of 10 L/min via a simple mask for approximately 10 days. Significantly, stabilization of oxygenation coincided with a decline in seizures and involuntary movements.

**Figure 1:**
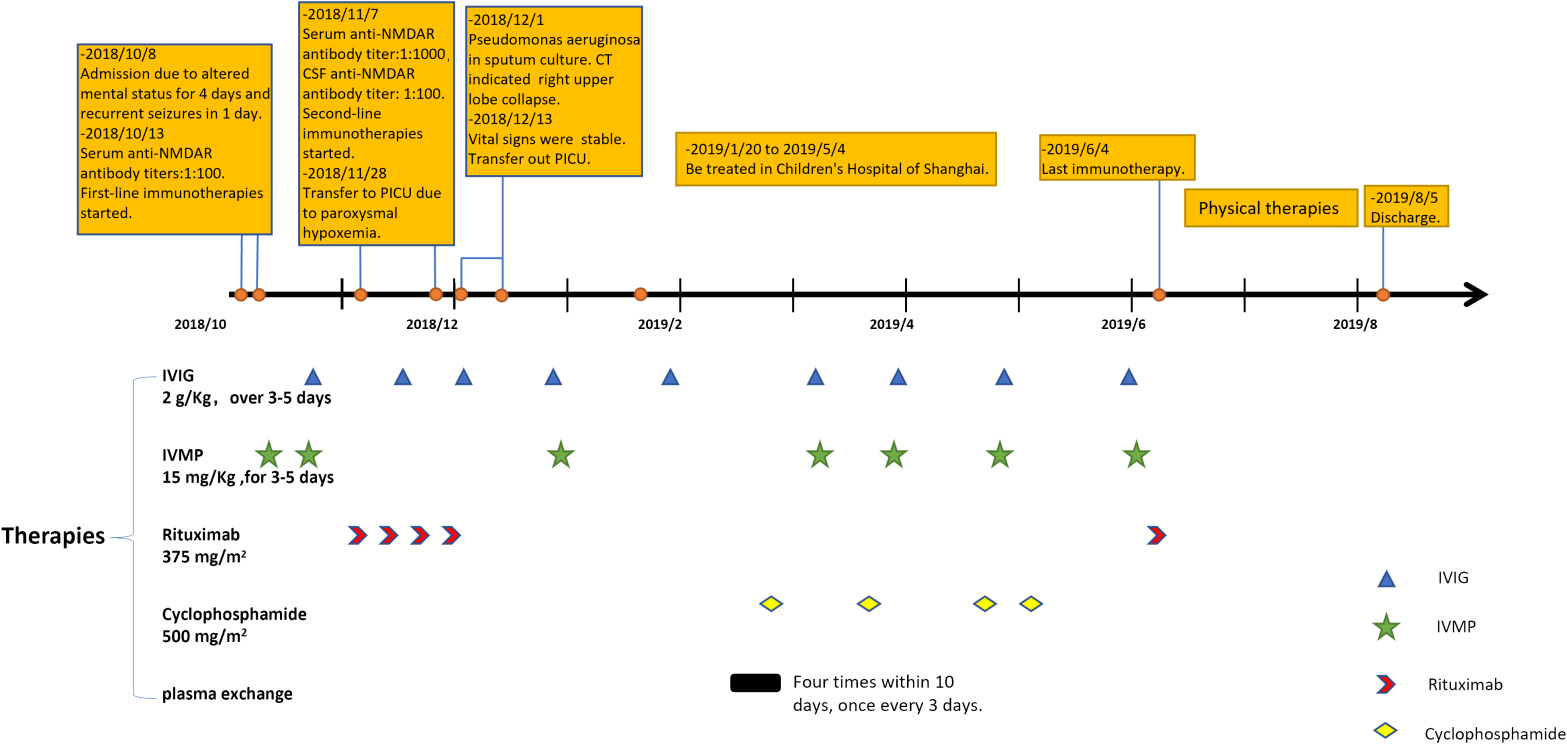
The timeline of our patient in 1 year after disease onset and her immunotherapies. IVIG, intravenous immunoglobulins; IVMP, intravenous methylprednisolone.

Despite receiving multiple immunotherapies, the patient remained unconscious, with a Glasgow Coma Scale (GCS) score of E4V2M4 (she could open eyes spontaneously and only make incomprehensible sounds with withdrawal from painful stimuli). After 3 months in our hospital, her parents opted to transfer her to the Children’s Hospital of Shanghai. There, she underwent additional rounds of immunotherapy including IVMP, IVIG, plasma exchange, and cyclophosphamide. By the sixth month of her illness (late April 2019), her eyes could follow objects and she could say certain words (for example, she said “dinosaur” when her mother showed her a dinosaur toy). However, her symptoms fluctuated in the subsequent month, prompting another round of immunotherapy, including IVMP, IVIG, and rituximab, which marked the conclusion of her immunotherapy regimen (all immunotherapies that the patient received are shown in **Figure 1**). Following this (June 2019), her overall condition slowly but steadily improved, and she commenced rehabilitation to restore her motor and cognitive function. Despite entering a phase of clinical recovery, her serum antibody titer continued to fluctuate between 1:1,000 and 1:3,200.

**Figure 2:**
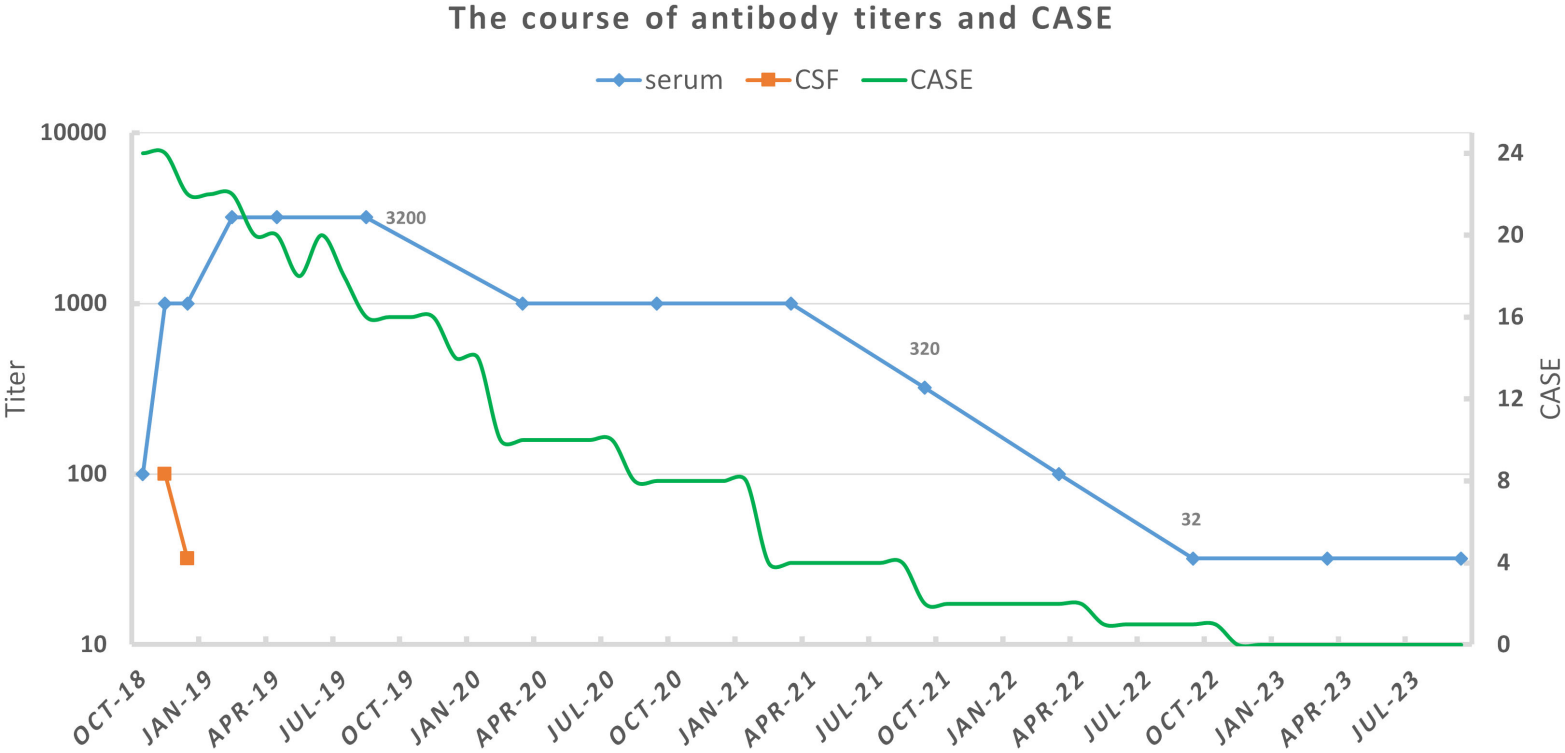
The course of antibody titers and clinical status for our patient. Antibody titers were tested by cell-based assay with HEK293 cells. Clinical status was assessed by Clinical Assessment Scale for Autoimmune Encephalitis (CASE) according to medical records and follow-up (6).

After her discharge in August 2019, she rechecked serum anti-NMDAR antibody titers every half a year, as well as gynecological ultrasound and chest x-ray annually. No sign of tumor was found during her follow-up. In the spring of 2020, even though she could not walk independently or speak fluently with a serum antibody titer of 1:1,000, she had returned to school and could get average scores on exams. By the spring of 2021, she could walk without assistance and engage in long conversation. The serum antibody titer decreased to 1:320 at that time. The titer further declined to 1:32 in November 2022, and remained until the latest test (31 January 2024). Immature behaviors were the main complaints of her parents in later years. However, she became more mature year by year in their eyes. Now, she has attended a key junior high school and exhibited behavior consistent with her pre-illness state, demonstrating good academic performance (the course of clinical status and antibody titers was exhibited in **Figure 2**).

## Discussion

While the anti-NMDAR antibody is among the most extensively researched and understood neuronal surface antibodies, its production and pathogenic mechanisms remain incompletely understood. Tumors and viral encephalitis are two confirmed triggers. Ovarian teratomas exhibit a notable association with anti-NMDAR encephalitis, although to a lesser extent in children. The presence of nervous tissues within ovarian teratomas may contribute to pathogenesis. Teratomas associated with anti-NMDAR encephalitis are significantly more likely to contain nervous tissues and exhibit greater inflammatory infiltrates of immune cells in tumor tissue (7). It is hypothesized that nervous tissues within teratomas may harbor NMDARs or similar structures exposed to infiltrating antigen-presenting cells such as B cells and tumor-associated dendritic cells. Regarding infection, the relationship between HSV encephalitis (HSE) and anti-NMDAR encephalitis has been extensively discussed, particularly in children (8). One prevailing theory suggests that HSV has a high affinity for limbic structures, leading to the release of neuronal antigens (9). There are also rare reports of other pathogens found in the CSF of anti-NMDAR encephalitis patients, including other types of herpesvirus, enterovirus, and adenovirus (8, 10). Anti-NMDAR antibodies could be present after HSE without corresponding symptoms (11). While infection may not directly induce antibody production, it likely plays a significant auxiliary role in facilitating antibody penetration across the blood–brain barrier (BBB) by increasing its permeability. Recently, studies on genetic predisposition in AE have yielded promising findings, with certain human leukocyte antigen (HLA) genes strongly associated with other forms of AE (12). Although no established HLAs have been linked to anti-NMDAR encephalitis, associations with HLA-A and HLA-DRB1 have been reported (13).

NMDAR antibodies typically induce cross-linking, internalization, and lysosomal degradation of NMDARs, resulting in the attenuation of NMDAR function. This effect is not complement-mediated and is generally reversible. Activation of the ephrin type B2 receptor can prevent this process (12, 14). Lynch et al. proposed that the antibody effect is dependent on NMDAR subtypes, where antibodies cause hyperfunction or hypofunction at extrasynaptic or synaptic NMDARs, respectively (14).

Rapid identification of anti-NMDAR encephalitis remains challenging, particularly due to atypical symptomatology in children. While psychiatric manifestations are often considered a red flag, they may hold less significance in children. This could be attributed to biological factors or technical limitations (15, 16). The psychopathological features of anti-NMDAR encephalitis are intricate, with some overlapping with neurological symptoms, particularly challenging in pediatric evaluations. Moreover, many pediatric practitioners may lack sufficient training in psychiatry, leading to potential misidentification of psychiatric phenotypes. Consequently, the incidence and phenotypes of anti-NMDAR encephalitis may vary significantly across studies. Catatonia is among the most frequently reported psychiatric syndromes in both adults and children, exhibiting a broad spectrum of symptoms. Chronic psychiatric and behavioral issues persist in approximately one-third of children for months to years following the onset of AE (17). However, the use of antipsychotic medications to manage psychiatric symptoms in AE should be approached cautiously, as these medications may exacerbate AE symptoms and increase the risk of neuroleptic malignant syndrome due to intolerance. Furthermore, antipsychotic medications often fail to alleviate symptoms (18, 19).

Seizures are among the major symptoms of anti-NMDAR encephalitis, and some individuals may progress to status epilepticus and require intensive care unit (ICU) admission, which are often predictive of a poor outcome (20). It is uncommon in children for seizures to occur as the sole manifestation. Generalized and focal seizures are nearly evenly distributed, with approximately half of all children presenting with both types of seizures (21). Seizures typically respond well to immunotherapy, particularly when compared to antibody-negative AE, which is associated with a lower rate of seizure freedom and a higher risk of postencephalitic seizures (22). The International League Against Epilepsy (ILAE) has proposed the term “acute symptomatic seizures secondary to autoimmune encephalitis” for seizures that resolve following immunotherapy, and “autoimmune-associated epilepsy” for seizures that persist (23). Levetiracetam and valproic acid are commonly used antiseizure medications in AE. Notably, dyskinesia is also a frequent presentation, posing challenges in differential diagnosis, especially in comatose patients. Comorbid catatonia–delirium, tonic–clonic seizures, and orolingual dyskinesia may serve as clinical hallmarks of anti-NMDAR encephalitis (24). In our patient’s case, significant paroxysmal orofacial automatisms were observed with no EEG correlations, and she consistently bit her buccal mucosa.

While the presence of antibodies is crucial for diagnosis, the testing process takes time and does not guarantee accuracy. Additionally, the existence of antibody-negative AE should not be overlooked. Diagnosis at onset is challenging, with epileptic seizures and sleep disturbances being two distinct features. EEG findings are often nonspecific, with extreme delta brush being a characteristic abnormality observed mainly in severely affected patients (25, 26). MRI has low sensitivity (less than 50%), and abnormal CSF findings are common but non-discriminatory compared to viral encephalitis. Detection of IgG intrathecal synthesis can enhance CSF sensitivity, with positive results more frequently seen in anti-NMDAR encephalitis than other types of AE (27, 28). Early identification heavily relies on recognizing initial symptoms. Although antibody detection is critical for diagnosis, commercial cell-based assays may yield false-negative results, necessitating brain immunohistochemistry if clinical suspicion remains high despite negative commercial assay results (29).

NMDAR antibodies are more readily detected in CSF than in serum, making it advisable to test for antibodies in both fluid compartments. Even with the use of three different techniques, Mar Guasp et al. found that 15% of patients with anti-NMDAR encephalitis did not exhibit antibodies in the serum but only in the CSF, a proportion that may be even higher in clinical practice. This discrepancy could be explained by activated T and B cells crossing the BBB and exclusively orchestrating the synthesis of NMDAR antibodies in the brain. Interestingly, patients with antibodies exclusively in the CSF appeared to have a milder form of the disease, although outcomes and relapse frequencies were similar to those with positive antibodies in both CSF and serum (30). As widely acknowledged, anti-NMDAR antibodies play a pivotal role in disease progression by reducing cell-surface and synaptic NMDAR levels, thus impairing memory and behavioral function (31). However, the ongoing controversy revolves around whether and how antibody titers correlate with clinical severity and prognosis. It is generally believed that CSF antibody titers hold more clinical significance than serum antibody titers (32). Observational studies have indicated that CSF antibody titers are positively correlated with ICU admission, ventilator use, and the presence of tumors (33). However, a study on hippocampal damage secondary to anti-NMDAR encephalitis found that neither CSF nor serum antibody titers corresponded with memory or cognitive performance (34). According to Cai et al., CSF antibody titers only exhibited a weak correlation with the modified Rankin scale (mRS) (*r* = 0.243) (35). A previous study by Xiaoting Zhang et al. demonstrated that CSF or serum antibody titers did not influence the response to first-line treatments (36). It is not uncommon for antibodies to persist in both serum and CSF, as evidenced by a retrospective study conducted by Gresa-Arribas et al., where 24 out of 28 CSF and 17 out of 23 serum samples from recovered patients remained antibody-positive (32). The prognostic value of persistent antibodies remains uncertain. A recent study found that patients with persistent CSF NMDAR antibodies at 12 months of follow-up experienced relapses more frequently and earlier. These patients also exhibited higher CSF antibody titers at diagnosis, longer ICU admissions, and received more extensive treatment. However, the same study did not find a significant difference in long-term outcomes between patients with persistent CSF antibodies and those without (37).

In our patient’s case, continuous monitoring of CSF antibodies was not feasible due to disagreement of her custodian. Before initiation of second-line immunotherapies, her CSF routine examination had turned normal. After using rituximab 375 mg/m^2^ weekly over a month, CSF antibody titers decreased from 1:100 to 1:32. Mitigation of seizures and dyskinesias was observed, especially after she was transferred from the PICU. It may be regarded as partial improvement, but also that could just result from the natural course of the disease (38). In contrast to CSF antibody titer, serum antibody titer significantly increased to 1:1,000 and then 1:3,200. Serum titers remained high even at the turning point, where she became responsive. They declined slowly and remained 1:32 after 5 years. That may indicate a sustained immune response. Refractory anti-NMDAR receptor encephalitis with high serum antibody titers had been reported in some cases. We list characteristics of some cases as well as ours in **Table 1**. All of them were female. Three had found teratomas and underwent teratoma resections. In a 14-year-old female patient, teratoma was found during follow-up (39). Existence of tumors is the most suspected reason for high serum antibody titers and treatment failure. One case underwent bilateral salpingo-oophorectomy due to consideration of non-visible teratomas, though she had tumor removal 1 year before (40). However, repeat abdominal and pelvic ultrasounds of our patient showed no abnormalities, which differed from those cases. We observed that no immediate titer drop happened after tumor resection. In the case of Serena et al., serum antibody titers rebounded during recovery with a titer of 1:800 (41). Unfortunately, they did not explain it. However, the report indicated that clinical status did not fluctuate. Moreover, it has been reported that infants born to mothers with anti-NMDAR encephalitis during pregnancy, and subsequently tested positive for the antibody, can exhibit normal growth and neuronal development (42). Our patient retained a high antibody titer of 1:3,200 when her general recovery began. Probably, serum anti-NMDAR antibodies alone would not cause symptoms or hinder recovery.

**Table 1:** Characteristics of patients with refractory anti-NMDAR encephalitis and high antibody titers.

Author	Thomas et al.	Tuhina et al.	Wang et al.	Serena et al.	Our case
**Age, sex**	Late 30s, F	14 years, F	27 years, F	29 years, F	8 years, F
**Onset**	Psychosis, headaches	Headaches, hallucinations, psychosis, seizures	Psychosis, seizures, loss of consciousness	Seizures, headaches	Altered mental status, seizures
**Tumor**	Teratoma	Teratoma	Teratoma	No	No
**MRI findings**	Normal	Abnormal	Abnormal	Abnormal	Normal
**Initial serum titer**	1:102,400	1:640	1:1,000	1:800	1:100
**Peak serum titer**	1:102,400	1:2,560	1:1,000	1:800	1:3,200
**Last serum titer**	1:25,600	1:160	1:300	1:800	1:32
**Follow-up period (since disease onset) (months)**	25	12	18	6	66
**Treatment**	Teratoma resection, IVMP, IVIG, RTX, CYC, MTX	IVMP, RTX, plasma exchange, bortezomib, teratoma resection	Teratoma resection, plasma exchange, RTX, IVMP, IVIG, CYC, intrathecal DXM and MTX	IVMP, IVIG, plasma exchange, RTX, CYC, bortezomib	IVMP, IVIG, plasma exchange, RTX, CYC
**Prognosis**	Bad	Good	Good	Good	Good

IVMP, intravenous methylprednisolone; IVIG, intravenous immunoglobulin; CTX, cyclophosphamide; RTX, rituximab; DXM, dexamethasone; MTX, methotrexate.

We were not certain what led to the recovery of our patient. Her recovery may be a result of previous treatments. Perhaps the effect of treatments may not be observed immediately. International consensus recommendations defined poorest responders as those who fail to make substantial and functionally useful improvements in the first 3 months after treatment commencement, but they also proposed that 18 months was the median overall duration of immunotherapy for the poorest responders (43). Still, no recommendations were given about cessation of immunotherapies. It was particularly challenging in severe patients like our case. Remarkably, our patient did not manifest any neuropsychological sequelae despite undergoing extensive treatment. A recent study on neuropsychological outcomes revealed common deficits such as lower sustained attention, worse verbal memory, and self-reported fatigue, none of which were observed in our patient after 5 years (44). Numerous studies have investigated predictors of outcomes, with early treatment initiation and avoidance of ICU admission identified as predictors of favorable outcomes in a systematic review (45). The One-Year Functional Status score (NEOS) has been proposed as a predictive tool for outcome at 1 year post-disease onset, with indicators including ICU admission, treatment delay exceeding 4 weeks, lack of clinical improvement within 4 weeks, abnormal MRI findings, and CSF white blood cell count exceeding 20 cells/μL (46). While these methods can aid in outcome prediction, they should be used cautiously to guide treatment decisions, along with consideration of persistent antibodies. To date, no significant biomarker has been identified to reliably guide treatment decisions (47). Therefore, as demonstrated by our case, clinical status remains paramount in treatment decisions.

Approximately one in five patients with anti-NMDAR encephalitis are refractory to standard first-line and second-line treatments. Recent reports suggest the efficacy of bortezomib in both adult and pediatric patients (48). Other agents such as mycophenolate mofetil, azathioprine, methotrexate, and tocilizumab have also been mentioned in previous years, but their use remains limited in scale with uncertain safety and efficacy (49, 50). Francesco Mannara et al. proposed allosteric modulation of NMDARs as a potential additional treatment. Their work demonstrated that SGE-301, a potent and selective positive allosteric modulator of NMDAR, prevented NMDAR dysfunction in a mouse model (51). Krienke et al. designed a noninflammatory messenger RNA vaccine delivering autoantigens into lymphoid dendritic cells in an experimental autoimmune encephalomyelitis model, resulting in antigen-specific tolerization and reduced disease severity (52).

In conclusion, pediatric anti-NMDAR encephalitis differs from the adult form in terms of pathophysiology and clinical manifestations. Early recognition and prompt initiation of immunotherapy are crucial for achieving a better prognosis. This case may instill confidence in clinicians, as despite prolonged impaired consciousness, lack of immediate response to first-line and second-line immunotherapies, and persistent serum antibodies after 5 years, the patient still experienced an unexpectedly positive outcome. We suggested that long-term prognosis and outcomes are generally favorable, even in cases where patients do not respond immediately to standard treatments and long-time unawareness occurs. Therefore, decisions regarding continuing immunotherapy should be based on clinical status rather than antibody titers, especially in serum. However, further studies with larger sample sizes are needed to thoroughly investigate the relationship between antibody titers, clinical severity, and prognosis.

## Patient’s perspective

The patient could not recall her illness properly, but she did not have any discomfort. Her parents described the event as “a long nightmare”, but was glad that they did not give up. At first, they were anxious about persistent antibodies in their daughter’s serum and the long-term prognosis of their daughter. However, their concerns had been alleviated since the patient recovered well. They hope to share their daughter’s experiences to inspire and help others suffering from the same disease.

## Data availability statement

The original contributions presented in the study are included in the article/supplementary material. Further inquiries can be directed to the corresponding author.

## Ethics statement

Written informed consent was obtained from the minor(s)’ legal guardian/next of kin for the publication of any potentially identifiable images or data included in this article.

## Author contributions

WZ: Writing – original draft, Writing – review & editing. WC: Writing – review & editing. WT: Writing – review & editing. YW: Writing – review & editing. CT: Writing – review & editing. QS: Writing – review & editing. XS: Supervision, Writing – review & editing.
